# An evaluation of contaminated complete feed as a vehicle for porcine epidemic diarrhea virus infection of naïve pigs following consumption via natural feeding behavior: proof of concept

**DOI:** 10.1186/s12917-014-0176-9

**Published:** 2014-08-05

**Authors:** Scott Dee, Travis Clement, Adam Schelkopf, Joel Nerem, David Knudsen, Jane Christopher-Hennings, Eric Nelson

**Affiliations:** 1 Pipestone Applied Research, Pipestone Veterinary Services, Pipestone, MN, USA; 2 Animal Disease Research and Diagnostic Laboratory, South Dakota State University, Brookings, SD, USA

**Keywords:** Porcine, Epidemic, Diarrhea, Virus, PEDV, Complete, Feed, Bioassay, Transmission

## Abstract

**Background:**

Since its initial detection in May 2013, porcine epidemic diarrhea virus (PEDV) has spread rapidly throughout the US swine industry. Initially, contaminated feed was proposed as a risk factor for PEDV; however, data were not available to support this theory. Here we provide proof of concept of this risk by describing a novel means for recovering PEDV-contaminated complete feed material from commercial swine sites and conducting an *in vivo* experiment to prove its infectivity.

**Results:**

For on-farm detection of PEDV RNA in feed, paint rollers were used to collect material from at-risk feed bins from 3 clinically affected breeding herds. This material was tested by PCR and determined to be positive for PEDV-RNA (Ct = 19.50-22.20 range). To test infectivity, this material was pooled (Ct = 20.65) and a Treatment group of 3-week old PEDV-naïve piglets were allowed to consume it via natural feeding behavior. For the purpose of a Positive control, piglets were allowed to ingest feed spiked with stock PEDV (Ct = 18.23) while the negative control group received PEDV-free feed. Clinical signs of PEDV infection (vomiting and diarrhea) and viral shedding were observed in both the Positive control and Treatment group’ post-consumption with virus and microscopic lesions detected in intestinal samples No evidence of infection was observed in the Negative controls.

**Conclusions:**

These data provide proof of concept that contaminated complete feed can serve as a vehicle for PEDV infection of naïve pigs using natural feeding behavior.

## Background

Porcine epidemic diarrhea virus (PEDV) is an enveloped single-stranded positive sense RNA virus belonging to the Order *Nidovirales*, the family *Coronaviridae* and the genus *Alphacoronavirus* (Saif et al. [1]). Following detection in the US swine population during May, 2013, the virus spread rapidly across the country and 6317 cases of Porcine Epidemic Diarrhea (PED) have been confirmed in 29 states as of May 3, 2014 [2],[3]. While little information is known regarding the routes of PEDV transmission between herds, potential risk factors include infected pigs, contaminated transport and PEDV-positive aerosols [4]–[6]. Recently, contaminated feedstuffs have been proposed as a route of PEDV transmission to naïve pigs but its current status is unclear [7]. While an initial report from the Canadian Food Inspection Agency indicated that consumption of PEDV-positive porcine blood plasma caused disease in pigs, a follow-up study could not demonstrate that the feed pellets (complete feed) containing the blood plasma in question were capable of causing disease [8],[9]. Despite this lack of evidence, dietary modifications to enhance the biosecurity of feed have been recommended to reduce this perceived risk [10]. As more data regarding the risk of PEDV transmission via complete feed are needed, we conducted a study to test the risk of PEDV-contaminated complete feed using a novel on-farm sampling method for virus detection in feed along with an *in vivo* experiment (swine bioassay) using at-risk feed material. The study was based on the hypothesis that contaminated complete feed can serve as a vehicle for PEDV infection of naïve swine.

## Methods

### Clinical history

During the period of January 9–13, 2014, clinical Porcine Epidemic Diarrhea was diagnosed in 3 breeding herds following acute outbreaks of anorexia, diarrhea and vomiting in isolated groups of sows. These herds were part of an organized system of commercial pork production; Farm A (4973 sows) was located in NW Iowa, while Farms B and C, 3390 sows and 3016 sows respectively, were located in SW Minnesota. All 3 herds emphasized strict biosecurity, using protocols previously validated to reduce the risk of PRRSV infection [11],[12]. Once a diagnosis of PEDV was confirmed, an investigation of each site was conducted to identify possible routes of viral entry. During the investigation, a consistent observation common to all 3 herds was noted. Specifically, from January 6–9, 2014, all 3 farms experienced an unexpected feed outage which required an “emergency” delivery. The emergency delivery had been deposited into a designated external storage bin which sourced feed to a distinct subpopulation of the herd. Following consumption of said feed, clinical signs became apparent only in the animals that had consumed this feed, i.e. no other signs were noted in other animals consuming other feed from other bins.

Based on this history, information regarding dates corresponding to recent feed deliveries, the location of the associated storage bin, the period of time between delivery of feed and clinical signs, the location of index cases in each farm, mill source and whether porcine by-products were present in feed was collected during the investigation. In addition, all transport-related activities, diagnostic data pertaining to recent genetic introductions, and records of personnel and supply entry to each farm were reviewed. Finally, as Farms A and B were air filtered, an evaluation of filter integrity and inspection for the potential of air bypass (entry of non-filtered air through improperly sealed fans, etc.) was conducted.

### Feed sampling

Because of the potential link to feed, it was planned to sample the designated bin on each farm to determine whether PEDV could be detected in “at-risk feed”, which was defined as feed consumed by the index population. Unfortunately, across all 3 sites, the majority of feed defined as “at-risk” had been consumed, leaving the designated bins nearly (or completely) empty. However, upon inspection of the bin lumen it was observed that clusters of feed material (feed particles and feed dust) were adhered to the interior walls. To access this material, a modification of a published method for sampling contaminated transport for PEDV RNA was devised [5]. Specifically, synthetic woven paint roller pads, 23 cm in length, 0.95 cm nap length (Sherwin Williams, Cleveland OH, USA) were attached to 3.6 m extension poles to access the cylindrical surface area of interior bin walls at multiple heights. To minimize environmental contamination of the roller prior to placement within the bin interior, a 4.4 L plastic bag (Ziploc, SC Johnson & Son Inc, Racine, WI, USA) covered the roller during ascension of the bin ladder. Following insertion of the roller into the bin lumen, the bag was removed and the roller was drawn across the inner walls, forcing large quantities of the adhered feed material to attach to the pad. In addition, if stored feed was present in a bin, the pad was drawn across the top layer to collect more material. Upon completion of sampling, the bag was replaced over the roller and the entire sampling apparatus was removed from the bin. Once on the ground, 200 mL of 7.2% phosphate buffered saline was poured into the bag, immersing the pad and promoting absorption of liquid. Using manual pressure, liquid was then forced from the pad into the bag and a 10 mL aliquot was decanted into a 15 mL plastic Falcon tube (Becton Dickenson, Franklin Lakes, NJ, USA) for diagnostic testing.

In addition to the sampling of the “at risk feed bin” on each farm, an “on-farm control bin” was also sampled. Control bins were located within 10 m of the “at-risk bin” but had not received a recent feed delivery and animals consuming feed from these bins were not clinically affected. Finally, to insure that the method did not generate false positive results, 8 feed bins across 4 PEDV negative farms were also sampled. All samples were tested for the presence of PEDV RNA using a RT-PCR at the South Dakota State University Animal Disease Research and Diagnostic Laboratory (SDSU ADRDL). A sample with a Cycle threshold (Ct) of less than 38 was considered PEDV positive.

### Swine bioassay facilities and source of animals

The swine bioassay component of this study was conducted in Biosafety Level 2+ rooms at the Animal Resource Wing (ARW) at SDSU. All procedures involving animals throughout the study were performed under the guidance and approval of the SDSU Institutional Animal Care and Use Committee. Animals (n = 11, three-week old piglets) were sourced from a PEDV-naïve herd and were tested on arrival to the ARW via blood sampling and collection of rectal swabs from each pig. Prior to animal arrival, all rooms (walls, ceilings, floors and drains) were monitored for the presence of PEDV by PCR using sampling procedures previously described (8). In addition, feed was sourced from a PEDV-naïve farm and screened by PCR prior to use.

### Experimental design

For the purpose of the swine bioassay, 11 piglets were divided into 3 groups and house each group in a pen within a designated room as follows:

**Treatment group:** 5 piglets to be fed challenge material consisting of the PCR-positive feed bin samples from herds A, B and C.

**Positive control group:** 4 piglets to be challenged with feed spiked with stock PEDV [13].

**Negative control group:** 2 piglets to be fed a placebo (feed + saline).

The study encompassed an 8-day period with challenge (consumption of designated feed material) occurring on day 0, followed by 6 days of diagnostic monitoring with necropsies conducted on day 7 post-challenge. Piglets were offered free-choice access to challenge material on day 0 of the study, allowing for natural feeding behavior, rather than to administer the challenge via gavage. Following IACUC approval, feed was withheld from all piglets for 12 hours prior to challenge. For the preparation of challenge for the Treatment group, 30 grams of feed material from the PCR-positive bin samples from Farms A, B and C was pooled and diluted in 30 mL of sterile phosphate buffered saline. The solution was vortexed for 2 minutes and then centrifuged at 16,000 g for 2 minutes. The supernatant was used in the PCR extraction and was then mixed with 454 grams of documented PEDV-free feed. In the case of the positive control group, 30 mL of stock PEDV was added to 454 grams of documented PEDV-free feed. Finally, for the Negative control group, an equivalent quantity of saline was added to 454 grams of PEDV-negative feed. Following consumption of challenge material, piglets were fed PEDV-free feed ad libitum for the remainder of the study.

### Piglet testing

Following consumption of their respective challenge material on day 0, the PEDV status in piglets across all 3 groups was monitored over time. On a daily basis, ARW personnel inspected animals for clinical signs of PED and collected samples as needed. Personnel moved from the Negative control group, to the Treatment group to the Positive control group every day. Showers were taken between rooms and room-specific coveralls, footwear, hairnets, gloves and P95 masks were worn. In addition, each room was ventilated individually, and HEPA filtration for both incoming and outgoing air was employed per room. If clinically affected animals were observed, rectal swabs (Dacron swabs, Fisher Scientific, Franklin Lakes, NJ, USA) were collected, along with swabs of any detectable diarrhea and vomiting. Swabs were submitted to the SDSU ADRDL and tested by PCR. On day 7 of the study, animals were humanely euthanized with intravenous sodium pentobarbital and intestinal tracts submitted for PCR and immunohistochemistry (IHC) testing and microscopic evaluation. Select samples were nucleic acid sequenced.

### Diagnostic procedures

All diagnostic testing was conducted using protocols developed and validated by the South Dakota State University Animal Disease Research and Diagnostic Laboratory.

### Polymerase chain reaction

#### Extraction of RNA

The MagMAX™ 96 Viral Isolation Kit (Life Technologies, Waltham MA, USA) kit was used to obtain viral RNA from the samples, as described in the instructions provided (1836 M Revision F). A 175-μl volume of sample was used for the extraction. The magnetic bead extractions were completed on a Kingfisher96 instrument (Thermo Scientific, Waltham MA, USA).

#### Real-time PCR

A commercially available real-time, single tube RT-PCR assay for the detection of PEDV and TGEV was used in this study per kit instruction (Tetracore, Rockville, MD, USA). Briefly, 7 μl of the extracted RNA was added to 18 μl of the master mix. The one-step real-time RT-PCR amplification conditions started with 15 minutes at 48°C, followed by 2 minutes at 95°C. The final cycles consisted of 5 seconds at 95°C and then 40 seconds at 60°C (data collection step). The program was run for 38 cycles (Cycle time) and the FAM detector was used for PEDV and the TAMRA detector was used for TGEV. Positive and negative controls were included on each run. All amplification was completed on the ABI7500 instrumentation (Austin, TX, USA).

### PEDV stock virus propagation

A cell-culture adapted variant of PEDV was used for inoculation of Positive controls. For PEDV propagation, Vero 76 cells (ATCC CRL-1587) were maintained in MEM plus 10% fetal bovine serum and antibiotics. Three-day old confluent monolayers of Vero 76 cells in 150 cm^2^ flasks were washed 3 times with serum free minimum essential media (MEM) prior to inoculation. Monolayers were infected at ~0.1 moi of PEDV in MEM containing 2.5ug/ml TPCK-treated trypsin, incubated at 37°C for approximately 48 hrs until obvious CPE was apparent. Flasks were frozen at −80°C until needed.

### PEDV S1 Sequencing

For select samples, it was planned to conduct nucleic acid sequencing of the PEDV S1 gene. Specifically, fragments of the S1 domain of the spike gene were amplified from extracted RNA. Primers **1:** (5′- ATGARGTCTTTAAYYTACTTCTGG-3′), **2:** (5′-CATCCTCACCWGCACTAGTAAC-3′), **3:** (5′- GTTGTGCTATGCAATATGTTTAY-3′), **4:** (5′-TGAAATTAATTGTGACAGCATC-3′), **5:** (5′ -TTGTCATCACCAAGTAYGGTG -3′), **6:** (5′- CTAAAAGACAGGTAATCATTAACAG- 3′), **7:** (5′- CTGTGTTGACACTAGACAATTTAC- 3′), **8:** (5′- CATACTAAAGTTGGTGGGAATAC- 3′) were designed to anneal to conserved genomic regions. Incorporation of degenerate bases maximizes the ability of the PCR to amplify genetically divergent PEDV variants between the US and UK strains. Primer pair 1 and 2 obtained a PCR product size of 670 bp, primer pair 3 and 4 obtained a PCR product size of 678 bp, primer pair 5 and 6 obtained a PCR product size of 565 bp and primer pair 7 and 8 obtained a PCR product size of 745 bp. Fragments were assembled sing the Vector NTI Software (Life Technologies, Waltham, MA, USA) for a complete S1 domain. QIAGEN One-Step Master Mix (Valencia, CA, USA) was used per kit instructions with an annealing temperature of 58°C for 30 seconds.

### Immunohistochemistry

Immunohistochemistry slides were prepared using the standard SDSU ADRDL IHC procedure, with the following modification being the use of PEDV Monoclonal antibody, of mouse ascites origin, courtesy of Steve Lawson, SDSU, at a 1:1000 dilution.

## Results

### Clinical history and feed sampling

During the on-farm inspection, no obvious breaches in any of the biosecurity protocols were detected across all 3 herds. No evidence of viral entry through genetic introduction, personnel error, contaminated transport or supplies were noted upon review of on-farm documentation. Finally, no breaches in the air filtration system (filter integrity, air bypass etc.) were noted on Farms A and B. In regards to feed, all herds received feed from different mills and diets contained corn, soybean meal, vitamins and trace minerals. No porcine by-products were included in any diet. Upon review of the history, a temporal relationship between the delivery of at-risk feed and the onset of clinical signs in index cases was observed (Table 1). Across all 3 herds, clinical signs were observed within 2 days post-delivery of at-risk feed. In addition, index cases were isolated to isolated areas of each farm which only received at-risk feed. Specifically, Farm A cases were located in the exterior row of stalls in the east gestation room and in gilts housed in a single room in the developer facility. For Farm B, index cases were located in the west farrowing room while the exterior row of stalls in the north gestation room housed index cases for Farms C. Assessment of feed material in the at-risk bins across the 3 affected sites indicated the presence of PEDV RNA with Ct values ranging from 19.50-22.20 (Table 1). In contrast, all samples from on-farm control bins and samples from bins on PEDV-negative sites were PCR-negative.

**Table 1:** Temporal relationship between the delivery of “at-risk” feed and the onset of clinical PED in the index cases across the 3 affected breeding herds

**Farm**	**A**	**B**	**C**
**Delivery of at risk feed**	January 6	January 8	January 9
**Date feed consumed**	January 6-7	January 8-9	January 10
Consumed by*	Gestating sows Developing gilts	Farrowing sows	Gestating sows
**PEDV Ct in feed**	20.25	22.20	19.50
**Onset of clinical signs**	January 9	January 10	January 12
Index cases*	Gestating sows Developing gilts	Farrowing sows	Gestating sows
**Date of PEDV diagnosis**	January 9	January 11	January 13

*Supplementary information: The same animals which consumed at-risk feed from the respective feed bins demonstrated clinical signs of PEDV. Specifically, affected gestation sows were located in exterior row of stalls in west gestation facility of Farm A while gilts were located in room 2 of the developer facility. Affected farrowing sows were located in the west lactation room of Farm B. Affected gestation sows were located in the exterior row of the north gestation facility in Farm C.

### Swine bioassay

**Figure 1:**
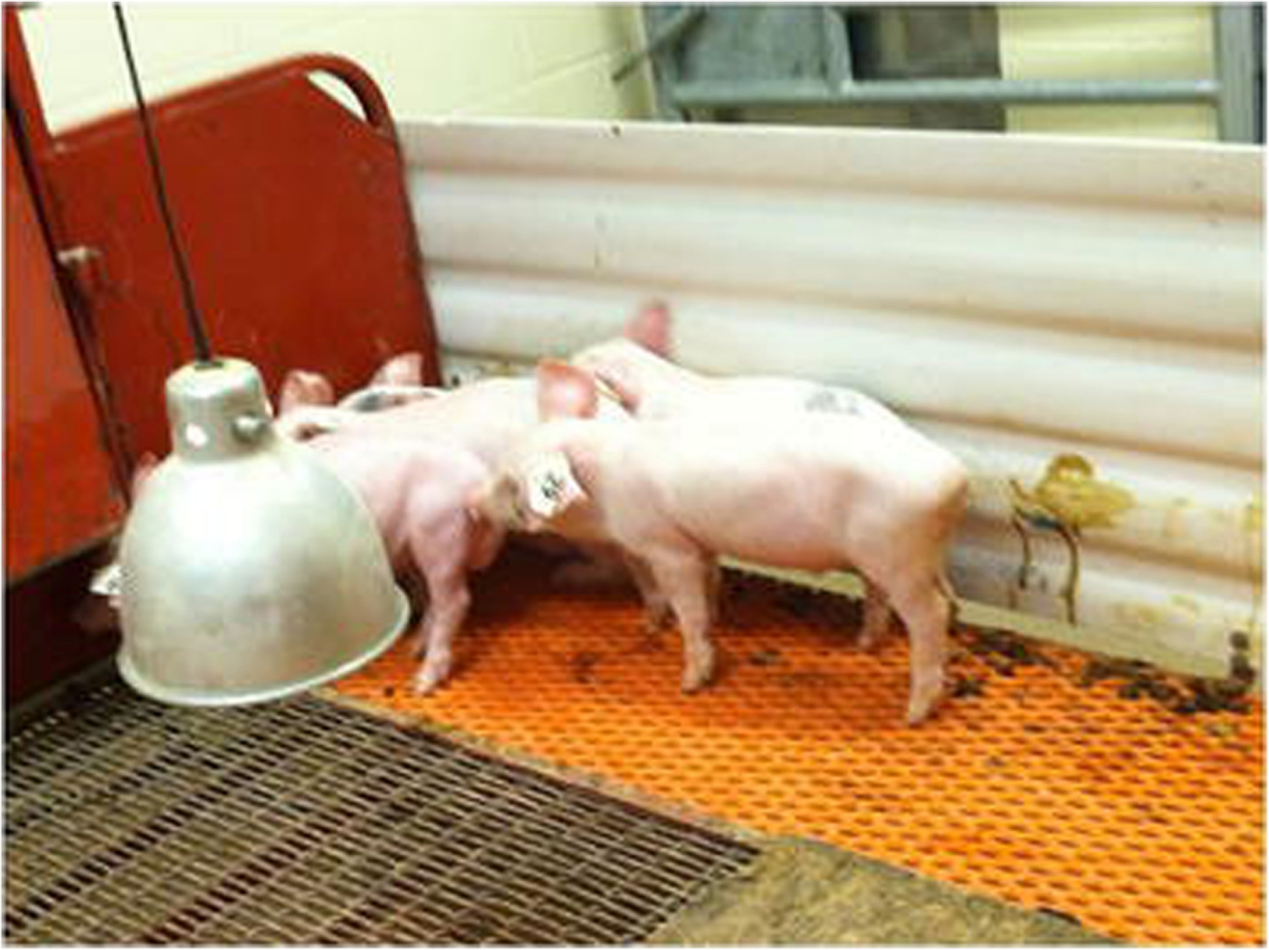
**PED in treatment group.** Depicted in this figure are clinically affected piglets from the Treatment group on day 6 post-ingestion of PEDV-contaminated feed. Piglets demonstrated loss of condition, rough hair coats, along with clinical signs of PED (diarrhea and vomiting). Evidence of diarrhea is visible on the pen wall behind the pigs. Prior to necropsy, rectal swabs from all 5 piglets were PEDV-positive as detected by PCR.

**Figure 2:**
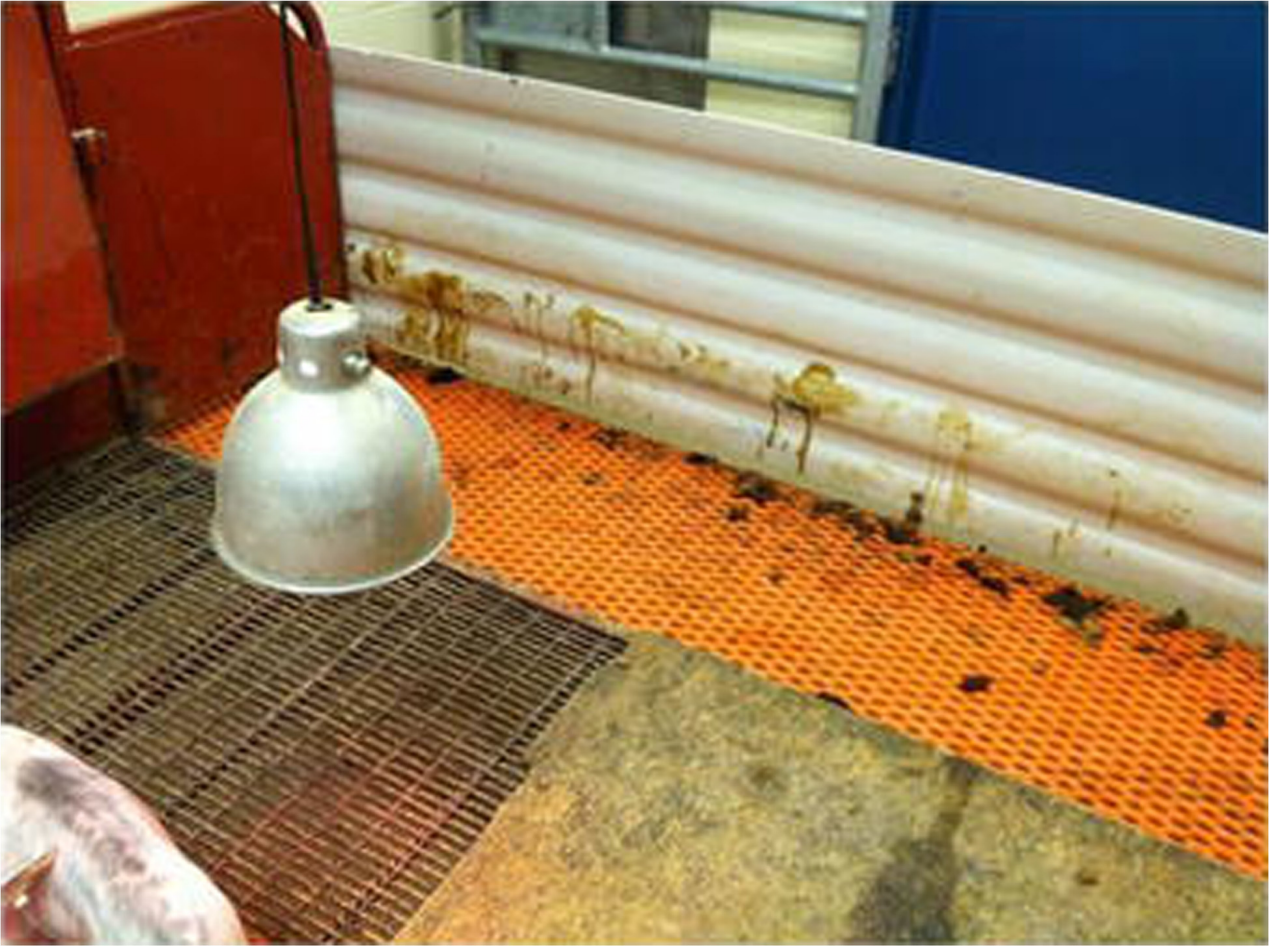
**Diarrhea in the treatment group pen.** This figure illustrates fecal staining on the wall of the pen housing the Treatment group piglets. This image was taken on day 6 post-ingestion of PEDV-positive feed.

**Figure 3:**
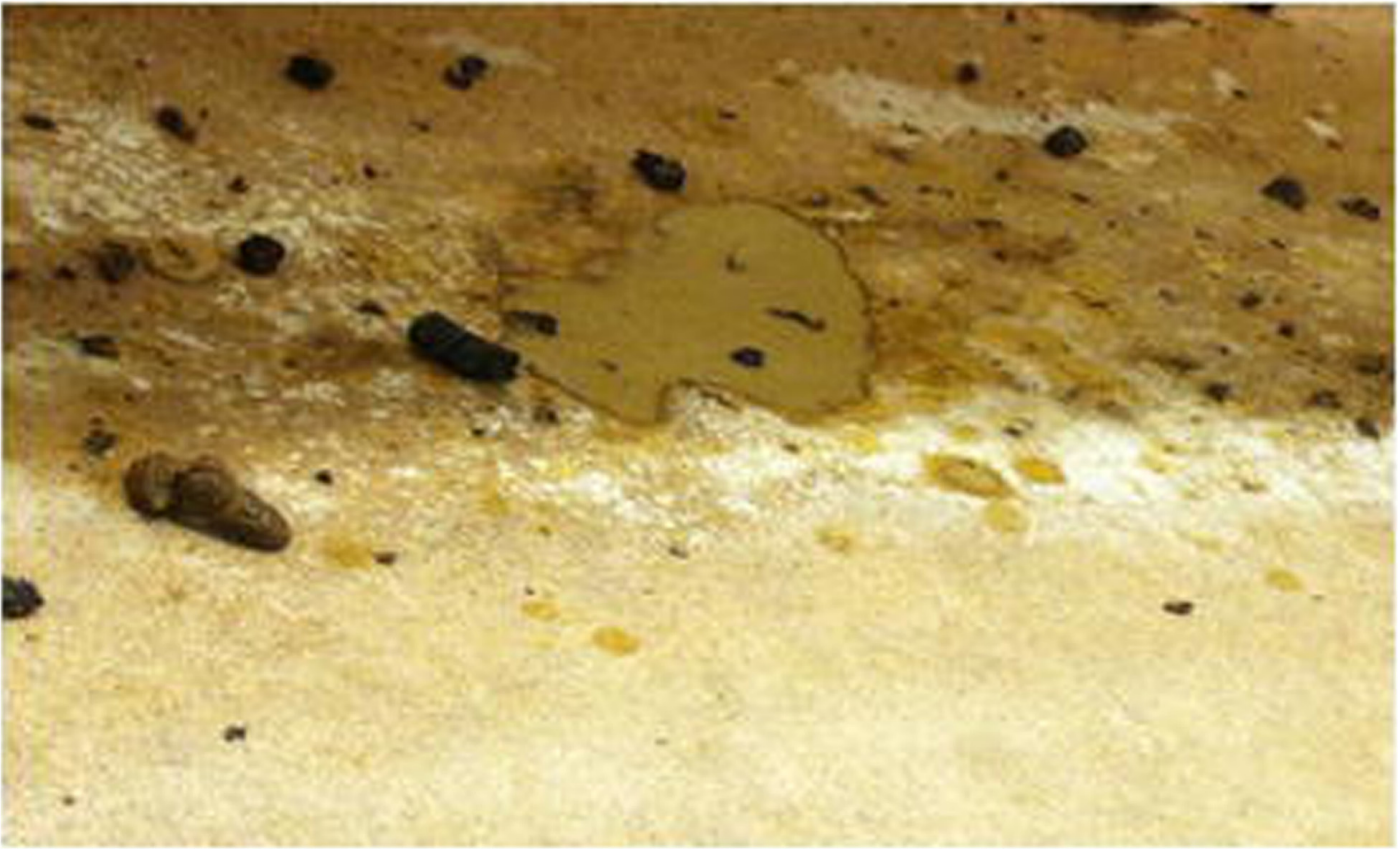
**Diarrhea on floor below the positive control group pen.** Following ingestion of feed spiked with stock PEDV, piglets in the Positive control group developed clinical signs of PED 2 days post-ingestion. This photo illustrates watery diarrhea observed below the pen floor housing these piglets.

**Figure 4:**
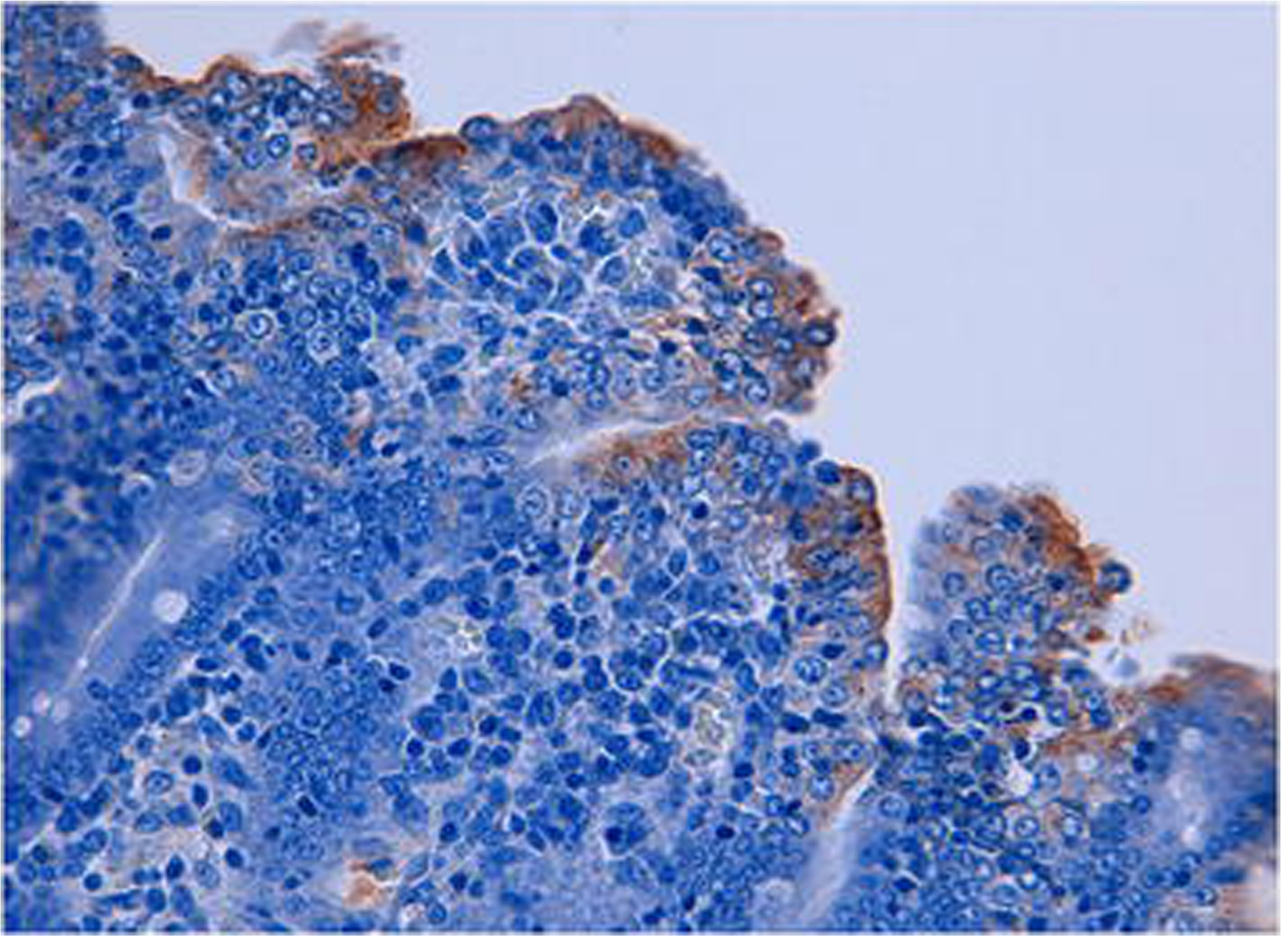
**Presence of PEDV antigen in a jejunal section from a treatment group piglet.** This photomicrograph (400x) illustrates the presence of PEDV antigen in multiple infected enterocytes following application of immunohistochemical staining.

**Figure 5:**
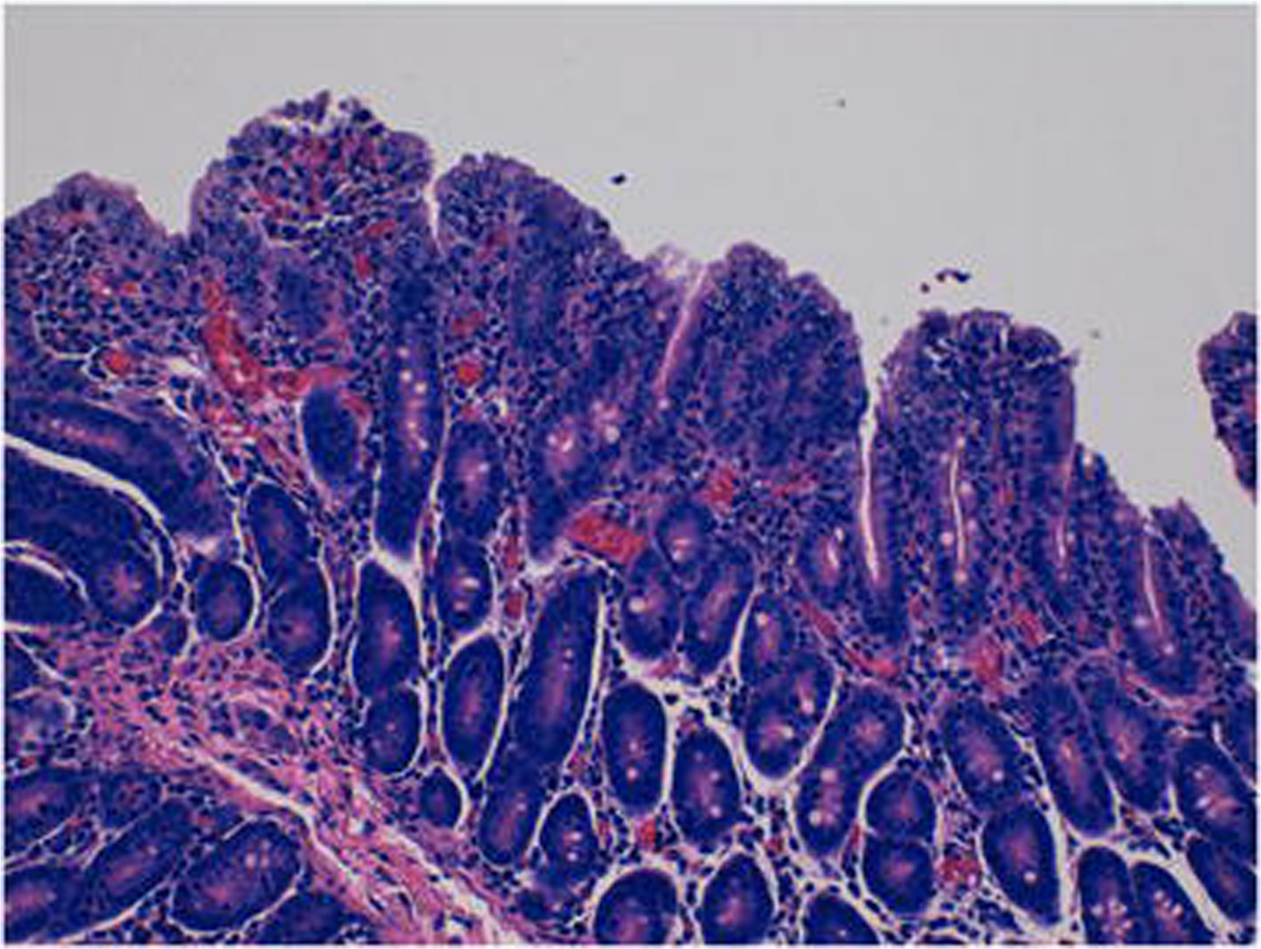
**Histopathological changes caused by PEDV.** This photomicrograph (400x) of a jejunal section from a Treatment group piglet demonstrates lesions secondary to PEDV infection, i.e. degeneration of enterocytes as indicated by the presence of re-epithelialization, along with villous blunting and fusion.

The in vivo phase of the study was conducted from January 17–24 and results are summarized in Table 2. Prior to initiation of the *in vivo* phase of the study, all samples from incoming piglets, ARW facilities and feed were PCR negative. In regards to the Treatment group, the pooling of feed material from Farms A, B and C resulted in challenge material having a Ct value of 20.65. Following challenge, clinical signs of diarrhea were observed in the index piglet in the Treatment group on day 4 post-ingestion. PEDV-RNA was detected in a rectal swab from this animal, along with swabs collected from diarrhea in the pen. This piglet continued to shed through the remainder of the study period and 1 other piglet displayed clinical signs of diarrhea and vomiting on day 6 post-ingestion (Figures 1, 2 and 3). Following euthanasia, rectal swabs and intestinal tract samples from all 5 pigs were positive by PCR and IHC (Figure 4). In addition, microscopic evaluation of small intestinal tissues indicated re-epithelialization with diffuse villous blunting and fusion was noted (Figure 5). In the Positive control group, the Ct of the stock virus used to spike its respective challenge feed was 18.23. Following consumption, shedding and clinical signs were observed in the index piglet on day 2 post-consumption with subsequent evidence of viral shedding to 2 other piglets on days 3 and 4. Similar to the Treatment group, all rectal swab samples and intestinal tract samples were PCR and IHC-positive at necropsy, along with evidence of microscopic lesions. In contrast, clinical signs, viral shedding or PEDV-positive intestinal tract samples were not observed in the Negative control group.

**Table 2:** Summary of clinical signs and diagnostic data across the 3 groups of pigs involved in the swine bioassay

		**Treatments**		**Positive controls**		**Negative controls**	
**Date**	**DPI**	**Rectal Ct**	**Clinical signs**	**Rectal Ct**	**Clinical signs**	**Rectal Ct**	**Clinical signs**
1/17	0	neg	neg	neg	neg	neg	neg
1/18	1	neg	neg	neg	neg	neg	neg
1/19	2	neg	neg	29.63	Vomit 32.21	neg	neg
1/20	3	neg	neg	16.06/32.21*	neg	neg	neg
1/21	4	34.09	Diarrhea 18.94	15.48/29.63*	Diarrhea 23.19	neg	neg
1/22	5	28.89	Diarrhea 16.23	15.79	neg	neg	neg
1/23	6	15.01/18.94*	Vomit 14.59	16.94	neg	neg	neg
1/24	Nx	5/5 pigs PCR/IHC (+)		4/4 pigs PCR/IHC (+)		2/2 pigs PCR/IHC (−)	

**Nx** = Necropsy, small intestinal tracts.

**DPI** = Days post-ingestion of at-risk or spiked feed.

**V/D** = vomiting & diarrhea.

* = 2 pigs detected as positive on rectal swabs.

S1 PEDV sequencing was completed on PCR-positive feed challenge material from both the Positive control group and the Treatment group, as well as an intestinal homogenate from the index piglet in the Treatment group. The sequencing results of the intestine and the Treatment feed material were similar, but different from the Positive control sample, indicating intestinal infection from consumption of the feed material.

## Discussion

The purpose of this paper was to provide proof of concept that complete feed that was contaminated with PEDV could act as a vehicle for infection of naïve pigs. To accomplish this goal, we developed a novel means of sampling at-risk feed material under controlled field conditions and conducted a swine bioassay to demonstrate transmission under controlled experimental conditions. Under the conditions of this study, we successfully detected PEDV RNA in complete feed material across all 3 sites and proved its infectivity using natural feeding behavior, a novel finding not yet reported. These results were strengthened through the inclusion of on-farm control feed bins and bins from naïve farms and the use of a Negative control group and natural feeding behavior during the in *vivo* phase. The sequencing data also suggest that the intestinal infection in the Treatment group resulted from ingestion of pooled feed material from the 3 affected herds.

An acknowledged limitation of the study was that the *in vivo* study was not designed to estimate the frequency of feed-related PEDV infections. These results were based on very small populations of pigs and cannot be extrapolated to today’s commercial farm conditions. However, the ability to complete a successful swine bioassay using a small number of animals cannot be ignored and the fact that our at-risk feed samples were collected from large commercial production sites adds to the credibility of this first attempt to investigate this risk factor. It was interesting to note the pattern of shedding (as detected by rectal swabs) in the Treatment and Positive control groups. Despite the fact we employed small numbers of animals, shedding was first detected in an index case and spread occurred throughout the group of piglets and differences were observed between the 2 groups. This suggests that when small groups are employed and/or field samples used for inoculation, the bioassay period may need to be extended to avoid the risk of false negative results.

In closing, this is the first publication providing proof of concept of the risk of PEDV-contaminated complete feed to naïve pigs. It was interesting to note that since neither feed source contained animal by-products, suggesting that contamination may have occurred post-processing, however, this was not proven. Further studies should focus on understanding the possibility of post-processing contamination as well as evaluating the ability of intervention strategies, i.e. the application of heat and pressure through the pelleting process and/or the inclusion of select feed additives which may have anti-viral effects, for reduction of this risk. Finally, it is the authors hope that the results from this study will assist in uniting the North American veterinary profession with the feed and swine industries as we collectively move forward in reducing the threat of this devastating transboundary disease.

## Conclusions

These data provide the initial proof of concept that contaminated complete feed can serve as a vehicle for PEDV infection of naïve pigs. Information from this study should be used to justify the need for further research towards the mitigation of said risk.

### Availability of supporting data

The data set(s) supporting the results of this article is included within the article.

## Abbreviations

PEDV: Porcine epidemic diarrhea virus

PED: Porcine epidemic diarrhea

Ct: Cycle threshold

PCR: Polymerase chain reaction

ARW: Animal resource wing

FAM: Fluorescein

TAMRA: Carboxytetramethylrhodamin

MEM: Minimal essential media

## Competing interests

The authors declare that they have no competing interests.

## Authors’ contributions

SD: Developed study, co-wrote paper. TC: Conducted molecular diagnostics, co-wrote paper. AS: Participated in on-farm collection. JN: Participated in on-farm collection. DK: Conducted pathological assessment of tissues and developed Figures 4–5. JCH: Provided critical review and revising of paper. EN: Provided virological expertise at the laboratory level and co-wrote paper. All authors read and approved the final manuscript.
